# Genetic diversity and variation of Chinese fir from Fujian province and Taiwan, China, based on ISSR markers

**DOI:** 10.1371/journal.pone.0175571

**Published:** 2017-04-13

**Authors:** Yu Chen, Zhuqing Peng, Chao Wu, Zhihui Ma, Guochang Ding, Guangqiu Cao, Shaoning Ruan, Sizu Lin

**Affiliations:** 1 College of Forestry, Fujian Agriculture and Forestry University, Fuzhou, Fujian, China; 2 State Forestry Administration Engineering Research Center of Chinese Fir, Fuzhou, Fujian, China; 3 Department of Nature, Fujian Museum, Fuzhou, Fujian, China; 4 College of Computer and Information Sciences, Fujian Agriculture and Forestry University, Fuzhou, Fujian, China; 5 College of Life Sciences, Fujian Agriculture and Forestry University, Fuzhou, Fujian, China; The National Orchid Conservation Center of China; The Orchid Conservation & Research Center of Shenzhen, CHINA

## Abstract

Genetic diversity and variation among 11 populations of Chinese fir from Fujian province and Taiwan were assessed using inter-simple sequence repeat (ISSR) markers to reveal the evolutionary relationship in their distribution range in this report. Analysis of genetic parameters of the different populations showed that populations in Fujian province exhibited a greater level of genetic diversity than did the populations in Taiwan. Compared to Taiwan populations, significant limited gene flow were observed among Fujian populations. An UPGMA cluster analysis showed that the most individuals of Taiwan populations formed a single cluster, whereas 6 discrete clusters were formed by each population from Fujian. All populations were divided into 3 main groups and that all 5 populations from Taiwan were gathered into a subgroup combined with 2 populations, Dehua and Liancheng, formed one of the 3 main groups, which indicated relative stronger relatedness. It is supported by a genetic structure analysis. All those results are suggesting different levels of genetic diversity and variation of Chinese fir between Fujian and Taiwan, and indicating different patterns of evolutionary process and local environmental adaption.

## Introduction

Taiwan, which is a typical continental island, is approximately 200 km east of Fujian province, China. Plants in Taiwan have a strong relationship with mainland China [1], whereas unique features exist in response to the geographic conditions present due to genetic divergence and evolutionary process. In the late Pliocene to early Pleistocene, the collapse of the Taiwan Strait caused the separation of Taiwan and mainland China [2], which led to a discontinuous distribution of plants that were once joined. Under the long-term impact of geographical isolation, the development of plants emphasized adaption to the local environment at the genome-wide level. The variation of alleles in the genome in response to endemic tolerance [3] gradually diverges because of different balancing selection [4] to fitting conditions in Taiwan and mainland China.

Chinese fir (*Cunninghamia lanceolata* (Lamb.) Hook.), a rapidly growing coniferous timber wood species, originated in the late Mesozoic Jurassic, was once widely distributed in Eurasia [5], but now only survives in southern mainland China, Taiwan and northern Vietnam. Investigations of the genetic diversity of Chinese fir have been reported since 1994. An allozyme technique was utilized and found the levels of genetic diversity should be concluded to be from diverse environments, the inbreeding system and long historical cultivation [6]. Comparisons of Chinese fir from mainland China to the closely related species *C*. *Konishii* (endemic species/subspecies of Taiwan) were carried out by several Taiwanese scholars. A phylogeographic investigation used noncoding spacer sequences of the cpDNA to reveal low differentiation between populations of Taiwan and China [7], and was supported by subsequent examination [8]. Moreover, 6 evolutionary lineages were found in *C*. *konishii* and the migration patterns proposed to be attributed to multiple factors such as events of long-distance dispersal indicated the derivation of *C*. *konishii* from Chinese fir [9].

Inter-simple sequence repeat (ISSR) analysis is a common technique for detecting the genetic variation and divergence. As a dominant marker ISSR has some disadvantages. A recently study reported that ISSR marker is less efficient than AFLP (Amplified Fragment Length Polymorphism, another type of molecular marker) for the lower degree of polymorphism [10]. However, it is still a widespread DNA marker for its many observable loci, abundant polymorphisms, credible discriminating information, and especially for low technical requirement and effective cost [10–12]. Considering its advantages, ISSR have been extensively used to detect genetic diversity and variation in many species, either alone [12–16] or in combination with other types of markers [17–19].

In this study, ISSR amplification was utilized to evaluate the genetic divergence of wild Chinese fir populations from Fujian province and the Taiwan area to assess the different levels of genetic variation, reveal the relationships among all populations, especially between Fujian and Taiwan groups, and infer the possibility of a different evolutionary process of Chinese fir created by the separated continent-island environment.

## Material and methods

There were no specific permissions requirement in this study for the non-protected species involvement and the minimal invasive sampling methods.

### Plant material

**Fig 1:**
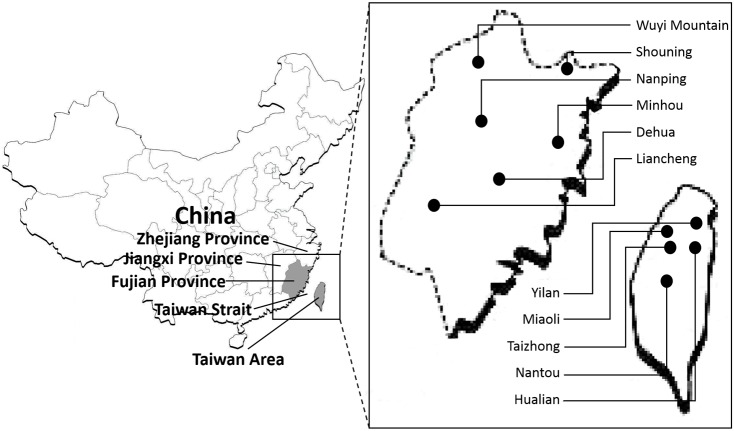
Fujian province and Taiwan area in China and the regions of the 11 populations that the Chinese fir materials came from.

To represent the distribution in Fujian province and the Taiwan area, 11 populations (6 populations in Fujian province and 5 in Taiwan), including 225 wild individuals of Chinese fir, were sampled and detected in this research (Fig 1). More details about the sampling locations are reported in Table 1.

**Table 1:** Details of the sampling locations including province/area, counties from where the populations were selected, sample size, latitudes, longitudes, altitudes and location description.

	Populations	Sample Size	Latitude (N)	Longitude (E)	Altitude (m)	Location
Fujian Province	Dehua	30	25°39′22.68″	118°08′06.36″	990	Daiyun Mountain Natural Reserve
	Liancheng	30	25°36′05.52″	116°56′07.26″	1160	Meihua Mountain Natural Reserve
	Wuyi Mountain	30	27°44′30.48″	117°42′04.68″	690	Wuyi Mountain Natural Reserve
	Shouning	30	27°25′59.88″	119°28′59.89″	600	Jingshan Forest District
	Nanping	30	26°39′02.94″	118°08′49.56″	330	Taiping Forest District
	Minhou	30	26°20′03.55″	119°41′11.76″	695	Minhou Village
Taiwan Area	Yilan	13	24°32′32.82″	121°23′03.93″	1960	Qilan Mountain
	Taizhong	14	24°08′25.00″	120°39′55.00″	2000	Danda, Siyuan, Dajia
	Miaoli	10	21°09′36.00″	120°32′24.00″	1700	Daan River
	Hualian	4	23°58′25.96″	120°58′55.29″	2220	Green Shenmu
	Nantou	4	23°38′30.18″	120°48′51.54″	2060	Xitou
	Total	225	-	-	-	-

Whereas confusion was brought by the large-scale seed source allocation in mainland China since 1978, a series of actions were used to ensure the regional characteristics could be represented. First, individuals from plantations were abandoned, and those from primary forests, natural reserves, and primitive villages were chosen. Second, age was estimated by diameter at breast height (DBH) and height to ensure that every wild individual tree was older than 35 years. In addition, the distance between any two individuals in a population was more than 1 km. For conservation purposes, cuttings shorter than 15 cm from each individual were brought to Fujian Agriculture and Forestry University to undergo cottage propagation and provide fresh young leaves for further experiments.

### DNA extraction and quantification

Total DNA was extracted from young fresh leaves using a CTAB (Cetyl trimethyl ammonium bromide) method [9]. The extract was treated with 5 μl RNase (10 mg/ml) at 37°C water bath for 1 hour to remove RNA [10]. To detect the quality of the DNA extract, 1% agarose gel electrophoresis was utilized. The concentration of the DNA extract was determined for each sample by using an ultraviolet spectrophotometer method.

### ISSR amplification

Sixty widespread ISSR primers from UBC (University of British Columbia, Vancouver, Canada) primer set #9 were used for this experiment. Those primers were synthesized by Shanghai Sangon Biological Inc., (Shanghai, China). A preliminary experiment using 2 donor samples as template was carried out to test the practicability of those primers. Based on the amplification bands that were clearly visible, polymorphic and stable, 18 primers (Table 2) were selected for further analysis.

**Table 2:** Profiles of the 18 ISSR primers including sequence, bands number, polymorphic bands number and polymorphic bands ratio.

Primer code	Sequence	Total bands number	Polymorphic bands number	Polymorphic bands ratio (%)
810	(GA)_8_T	12	9	75.0
812	(GA)_8_A	12	10	83.3
814	(CT)_8_A	16	8	50.0
815	(CT)_8_G	13	7	53.8
817	(CA)_8_A	13	9	69.2
820	(GT)_8_C	12	8	66.7
825	(AC)_8_T	13	11	84.6
827	(AC)_8_G	18	5	27.8
834	(AG)_8_YT	14	10	71.4
842	(GA)_8_YG	16	13	81.3
845	(CT)_8_RG	15	6	40.0
847	(CA)_8_RC	19	12	63.2
848	(CA)_8_RG	20	6	30.0
849	(GT)_8_YA	13	5	38.5
853	(TC)_8_RT	12	5	41.7
855	(AC)_8_YT	15	9	60.0
857	(AC)_8_YG	20	12	60.0
858	(TG)_8_RT	13	9	69.2
Total	-	266	154	57.9

The ISSR amplifications were carried out in 20 μl reactions which containing 2 μl 10×PCR buffer (without Mg^2+^), 1.5 mM Mg^2+^, 0.15 mM dNTPs, 0.4 μM primers, 1.5 U of TaqDNA polymerase and 75ng of template DNA. The reaction amplification program was: an initial denaturation of 5 min at 94°C, followed by 40 cycles of 45s at 94°C, 50s at 52°C, 90s at 72°C, and final extension at 72°C for 8 min. The PCR amplification products were separated by agarose gel electrophoresis, and all of the clear bands were counted for the subsequent analysis.

### Data collection and analysis

The *AlphaImager HP* version 1.0 software combined with an artificial counting method were utilized to score all of the polymorphic bands for the presence/absence of all genetic loci. The *POPGENE* version 1.32 software was utilized to estimate the genetic diversity among populations including the following parameters: observed number of alleles (*Na*), effective number of alleles (*Ne*), Nei’s gene diversity (*h*), Shannon’s information index (*I*) and genetic distance (*D*) [20]. The *ARLEQUIN* version 3.5 software was utilized to estimate of gene flow (*Nm*). A dendrogram was drawn using UPGMA clustering to describe the relatedness of all 11 populations, and a 1000 times bootstrap test was carried out to verify the result of that clustering. The *STRUCTURE* version 2.3.4 software was applied to obtain the hierarchical organization of the genetic structure of the 11 populations and the admixture model was selected as the ancestry model because of the high heterozygous rate of Chinese fir. The number of genetic groups (*K*) was determined referring to Evanno’s method [21,22]. The *MANTEL* function from *VEGAN* package of *R-STUDIO* version 0.98.1091 software was utilized to carry out a mantel test to check the correlation of the matrixes of genetic and geographic distances.

## Results

### Genetic diversity of Chinese fir in Fujian and Taiwan

Genetic diversity analysis using 60 widespread ISSR primers was carried out. Eighteen ISSR primers generating clear, identifiable and polymorphic bands were selected and totally 266 polymorphic loci were generated from those primers. All the present/absent information of the polymorphic loci were listed in the S1 Text file. The profiles of those primers (Table 2) showed that the mean percentage of polymorphic loci was 57.9% (ranged from 27.8% to 83.3%).

The main genetic parameters were reported in Table 3. The polymorphic allele numbers in the populations from Fujian province ranged from 204 to 220, whereas in the populations from Taiwan, they ranged from 112 to 207. The *h* parameter ranged from 0.207 to 0.243 (group level = 0.325) in Fujian province’s populations and from 0.157 to 0.278 (group level = 0.263) in the Taiwanese area’s populations; meanwhile, the *I* value ranged from 0.321 to 0.374 (group level = 0.494) in populations from Fujian province and from 0.233 to 0.411 (group level = 0.410) in Taiwan, which indicated the probability of higher genetic diversity among Chinese fir in Fujian province. At the population level, the Nanping population (*h* = 0.243; *I* = 0.374) and Miaoli population (*h* = 0.278; *I* = 0.411) had the highest degrees of genetic diversity. The low *Na* (range from 1.767 to 1.823, group level = 1.996 in Fujian province; range from 1.421 to 1.778, group level = 1.90 in Taiwan) and *Ne* (range from 1.340 to 1.400, group level = 1.538 in Fujian province; range from 1.271 to 1.495, group level = 1.425 in Taiwan) values identified in this experiment reflected a number of recessive mutation loci that could not be detected by ISSR markers as the dominant markers they are.

**Table 3:** Main genetic parameters of different populations.

Populations	Polymorphism alleles number	Polymorphic ratio (%)	*Na*	*Ne*	*h*	*I*
Dehua	210	78.95	1.790	1.351	0.216	0.336
Liancheng	212	79.70	1.797	1.359	0.219	0.341
Wuyi Mountain	213	80.08	1.801	1.364	0.223	0.346
Shouning	220	82.71	1.827	1.374	0.230	0.357
Nanping	219	82.33	1.823	1.400	0.243	0.374
Minhou	204	76.69	1.767	1.340	0.207	0.321
**Fujian group**	**-**	**-**	**1.996**	**1.538**	**0.325**	**0.494**
Yilan	186	69.92	1.699	1.342	0.205	0.315
Taizhong	168	63.16	1.632	1.333	0.198	0.300
Miaoli	207	77.82	1.778	1.495	0.278	0.411
Hualian	151	56.77	1.568	1.373	0.212	0.314
Nantou	112	42.11	1.421	1.271	0.157	0.233
**Taiwan group**	**-**	**-**	**1.970**	**1.425**	**0.263**	**0.410**

To ascertain the gene flow among the populations, the *Nm* value was calculated and is reported in Table 4. The result showed different levels of gene flow among those populations. The *Nms* within populations from Taiwan (range from 1.843 to 14.151) are significantly higher than within Fujian (range from 0.343 to 0.716) and between populations from Fujian and Taiwan (range from 0.308 to 0.575), which indicated a stronger connection within populations from the Taiwan group. The strongest gene flow was found between Hualian and Nantou populations. However, the signal of gene flow may possibly be amplified by the limited sample sizes from the both populations. The Miaoli population exhibited a relatively stronger gene flow to other populations: its *Nms* range from 0.492 to 7.664, which was slightly higher than the other populations, thus partly confirming the high rate of diversity as analyzed by the genetic parameters.

**Table 4:** Gene flow (*Nm*) among 11 populations.

Populations	Dehua	Liancheng	Wuyi Mountain	Shouning	Nanping	Minhou	Yilan	Taizhong	Miaoli	Hualian	Nantou
Dehua											
Liancheng	0.716										
Wuyi Mountain	0.431	0.454									
Shouning	0.516	0.436	0.440								
Nanping	0.439	0.403	0.365	0.563							
Minhou	0.355	0.365	0.343	0.379	0.341						
Yilan	0.442	0.468	0.369	0.475	0.444	0.360					
Taizhong	0.399	0.398	0.324	0.401	0.408	0.308	2.682				
Miaoli	0.569	0.575	0.492	0.553	0.541	0.505	2.955	2.259			
Hualian	0.422	0.445	0.393	0.468	0.437	0.405	2.564	1.843	7.664		
Nantou	0.407	0.388	0.370	0.428	0.444	0.347	2.465	1.660	3.906	14.151	

### Genetic relationship and structure

**Fig 2:**
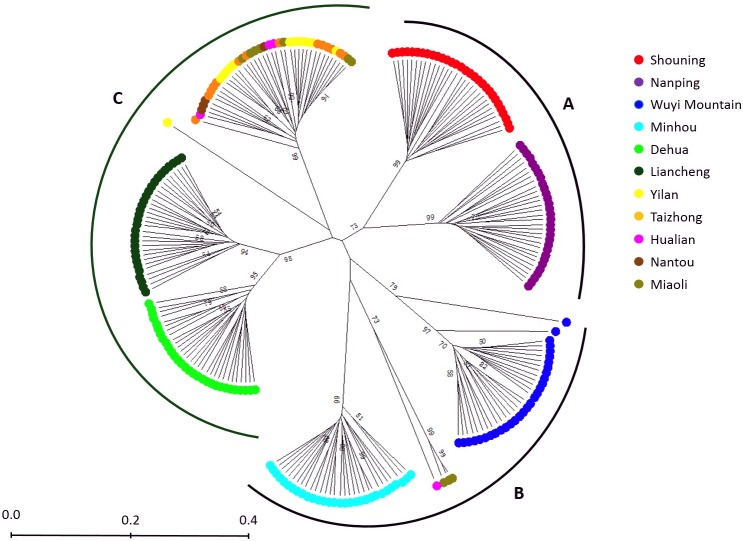
Dendrogram of genetic relatedness of 11 populations from Fujian province and Taiwan. Different populations were colored and distributed into 3 main clusters, 7 sub-clusters. Frequency values above 50 were displayed.

Cluster analysis and the UPGMA algorithm were used to generate a dendrogram, and the result is reported in Fig 2. According to the genetic distance, 7 clear clusters emerged. The 6 populations from Fujian province formed 6 discrete clusters. Whereas 4 individuals from the Miaoli and Hualian populations were closer to the Minhou population, most individuals of the 5 Taiwan area populations merged together and formed a single cluster. Three main clusters, A, B and C, could be obtained as long as the predicting genetic distance was increased for a larger view. Cluster A contain 2 sub-clusters (Shouning and Nanping), cluster B contain 2 sub-clusters (Wuyi Mountain and Minhou) and 4 individuals from Taiwan area, and cluster C contain 3 sub-clusters (Dehua, Liancheng, and Taiwan). Thus, Chinese fir in Taiwan may have stronger relatedness to those 2 populations.

**Fig 3:**
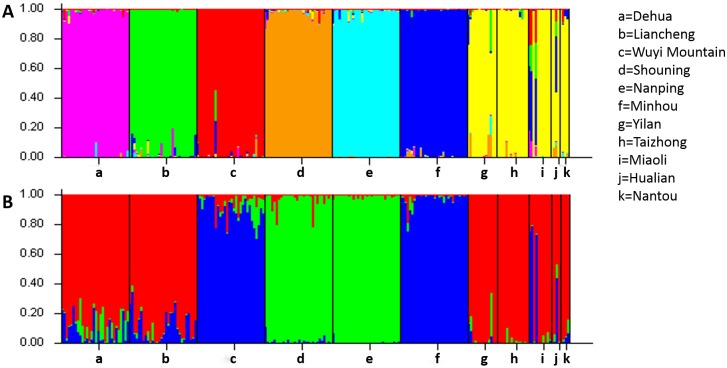
**A** Hierarchical organization of the genetic structure of 11 populations from Fujian province and the Taiwan area. Each letter represents one population as marked at the right part of the map. Different colors means different groups and the number of groups (*K*) was set to 7 based on the method of Evanno. The length of the colored segment suggested the estimated membership proportion of each sample in the designed group. **B** A similar organization of the genetic structure histogram difference lies in *K* = 3.

To estimate the reliability of the likely cluster groups analyzed already, the genetic structure of the 11 populations was calculated using the *STRUCTURE* software. The number of genetic groups (*K*) showed a visible peak at 7 (S1 Fig) by Evanno’s method [21], indicating that 7 groups should be distributed across all 11 populations (Fig 3A). Similar to the result of the cluster analysis, the 6 populations from Fujian province formed 6 distinguishable groups, whereas the populations from Taiwan area formed a single group. Based on the genetic structure, a rare hybridization event among Fujian Province was observed. Some individuals in Taiwan contained parts of genetic components that suggested the probability of hybridization among those populations. For further analysis, another structure calculation was carried out by setting the *K* value to 3. Similar to the result of the cluster analysis, the histogram showed 3 main groups (Fig 3B). The Dehua population, Liancheng population and populations from Taiwan formed a group; the Wuyi Mountain population and Minhou population formed another; and the Shouning population and Nanping population formed the third group. Thus, these results closely mirrored the pattern of diversity described in the UPGMA dendrogram.

### Genetic distance and geographical distance

The genetic distance between every 2 populations (Table 5, below diagonal) ranged from 0.033 (between Yilan population and Taizhong population) to 0.256 (between Wuyi Mountain population and Taizhong population). A clear phenomenon could be described in which the genetic distance inside of Taiwan (range from 0.033 to 0.113 with a mean = 0.071) was significantly shorter than the genetic distance inside of Fujian province (range from 0.090 to 0.212 with a mean = 0.167) or between populations from Fujian province and Taiwan (range from 0.125 to 0.256 with a mean = 0.193).

**Table 5:** Genetic distance (below diagonal) and geographical distance (above diagonal) of 11 populations, km.

Populations	Dehua	Liancheng	Wuyi Mountain	Shouning	Nanping	Minhou	Yilan	Taizhong	Miaoli	Hualian	Nantou
Dehua		133.46	118.67	177.93	52.43	109.97	366.59	293.59	278.81	328.27	316.34
Liancheng	0.090		138.26	299.44	143.93	240.28	498.16	422.47	407.54	456.98	442.67
Wuyi Mountain	0.162	0.158		198.94	74.18	169.02	445.43	383.17	369.34	417.02	410.24
Shouning	0.135	0.168	0.177		155.87	75.66	267.00	226.07	215.91	254.62	257.72
Nanping	0.155	0.182	0.218	0.129		105.14	379.75	313.35	299.14	247.65	339.26
Minhou	0.174	0.170	0.203	0.181	0.196		276.58	215.60	202.30	248.89	244.07
Yilan	0.161	0.153	0.212	0.170	0.173	0.188		82.92	96.81	56.66	82.93
Taizhong	0.189	0.193	0.256	0.213	0.198	0.230	0.033		14.95	34.68	32.50
Miaoli	0.129	0.125	0.154	0.157	0.158	0.12	0.073	0.096		49.51	42.36
Hualian	0.203	0.201	0.239	0.218	0.224	0.185	0.061	0.071	0.077		26.56
Nantou	0.213	0.229	0.254	0.225	0.204	0.210	0.058	0.065	0.113	0.063	

**Fig 4:**
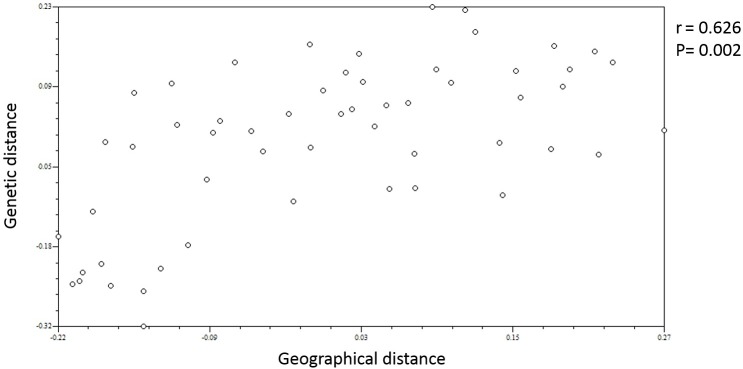
*MANTEL* test plots of the genetic distance and geographical distance. The vertical axis represents the genetic distance, and the abscissa axis represents the geographical distance. Both axes were standardized.

Based on the geographical distance (Table 5, above diagonal), the phenomenon described above for the genetic distance seems to be attributable to the location of the populations. The geographical distance inside Taiwan ranges from 14.95 km to 96.81 km, with a mean of 51.99 km; it ranges from 52.43 km to 299.44 km, with a mean of 146.21 km, inside Fujian province; and it ranges from 202.30 km to 498.16 km, with a mean of 327.48 km between populations from the two areas. A *MANTEL* test also supports this deduction (Fig 4, r = 0.626, P = 0.002).

## Discussion

Coniferous species are known for the conserved genome structure and slow evolutionary strategy to fit the nature selection [23,24]. A report by Loverless and Hamrick, which suggested a low average level of genetic diversity (*h* = 0.207) in coniferous species [25], provided an evidence for their low degree of genetic variation. However, there are some conifers, such as *S*. *verticillata* (*h* varied from 0.282 to 0.450) [26] and *N*. *longibracteata* (*h* varied from 0.291 to 0.851) [27], displaying relatively higher level of genetic diversity, which reflecting the long-term divergence by different local environmental conditions or climate change occurred in the coniferous species. Compared to a previous survey of Chinese fir, which focused on 16 populations from 9 provinces in mainland China (*h* = 0.394) [6], lower levels of genetic diversity were found in this present study both from Fujian province (*h* = 0.325) and Taiwan area (*h* = 0.263). The different levels of diversity were probably due to the locations of the sample populations. A long distance, which is obviously greater between provinces than within a province, could affect the gene flow by seed dispersal. Similar levels of genetic diversity were reported by other researches [28,29], suggested a medium degree genetic diversity of Chinese fir in species level.

The geographical isolation of continental islands usually provides systems to study the evolutionary process [30,31]. Nevertheless, the geographical barriers between islands and the nearby continents may not be consistently present since of the alteration of sea level during glacial periods. That provides an opportunity to investigate the models for the formation of the differentiation between continental island endemics and the relative populations in the nearby continent through the intermittent gene flow among the once-isolated populations [30]. As a continental island, Taiwan has been isolated from mainland China for a long period, and the species in Taiwan may vary gradually along different evolutionary processes. Similar to *C*. *Konishii* [9], *A*. *kawakamii* [32] and *M*. *thunbergii* [33], small genetic divergence (Table 3) and high degree of gene flow (Tale 4) of Chinese fir were found in Taiwan populations in this study, which rejected a restricted genetic exchange within Taiwan populations and supported a limited gene flow between Taiwan and Fujian. Additionally, the single group merged by populations from Taiwan and, in contrast, multiple groups formed by each population from Fujian (Fig 3A) also emphasized the differentiation between Taiwan and Fujian, implied the long-history of separation, which due to a limited number of landraces from mainland China were presumably introduced in Taiwan, and a possible recolonization in Taiwan by a small number of individuals after an extinct event which might occur during the glacial period.

Interestingly, an opposite pattern characterized by high genetic diversity (Table 3), population-specific clusters (Fig 2) and distinct genetic structure (Fig 3A) was found within Fujian populations, which inferring those populations may have been isolated for some time. The low level of gene flow, which usually illustrates a tendency of genetically homogeneous at the population level [34], further represented the possibility of ancient polymorphisms retained among the genetic and geographic isolates of today [35]. It received supports from investigations of *F*. *hodginsii*, which was also showed a high level of genetic diversity and low level of gene flow among populations in Fujian province [36], implied that it has not undergone an extinct event [37] in Fujian for the existence of a potential refugium [27,38,39].

To explain the different levels of genetic diversity between Fujian and Taiwan, a continent-island pattern for coniferous populations in evolution [40] could be used. ‘Sea level’ is supposed to produce different pressures on natural selection that forces colonization toward higher altitude. Populations located at different elevations were motivated by different levels of potential adaptation to the local environment [41,42], leading to the occurrence of loci diverging. Limited by the number of the populations and the size of the populations plus due to some types of bottleneck events [43], expansion of the populations upon one species was not always observed. A decrease in the distribution range of *A*. *kawakamii* [32] and *K*. *daviana var*. *formosana* [44], two pinaceous species in Taiwan, has been reported. In the present study, higher altitude of sampling locations in Taiwan area (Table 1) could probably create a more stringent selection pressure, and promote a potential extinct event, whereas the lower altitude in Fujian might offer a milder condition to Chinese fir, and protect the formation of the endemic. To a species that conquered the local environment and successfully increased its population number, extension into the hinterland of the island means a larger geographical distance from the original continent enhanced its spatial isolation and restricted the gene flow from outside. Isolation-by-distance has been observed in this study (Fig 4) may indicate that the effect would form a unique evolutionary process characterized by the unique environment of the island [45,46]. It is supported by some morphological differences of Chinese fir which were found in Taiwan populations [47].

Geological evidence has revealed that several small patches across the southern part of China and Taiwan provided some possible migration routes [7], such as the Ancient Route Way from central Fujian to Taizhong, and the Dongshan Overbridge [48], for many coniferous species to avoid extinction events and scatter into refuges during glacial periods. The present study revealed multiple sources of Chinese fir introduction from Fujian province to Taiwan, which has been proposed [7,9], by the UPGMA analysis (Fig 2). Two major groups, group B and C, were corresponding with the individuals from different regions. Group C shows a closer relatedness between most of the individuals from Taiwan and Dehua and Liancheng populations, both of which are located in the southern part of Fujian province compared to the other populations (Fig 1) and happened to be near the Dongshan Overbridge mentioned above, suggested a potential for a provenance relation of Chinese fir between Fujian province and Taiwan. However, it could be noticed that some individuals from Taiwan in group B contained genetic components of Wuyi Mountain and Minhou populations (Fig 3) might underline the agreement of a potential original region from north of Fujian. Pollen of Chinese fir was not found in unearthed fossils from the Pleistocene stratum in Jiangxi Province but was found in fossils from late Pleistocene to Holocene strata in coastal areas in Zhejiang province [49], which is adjacent to northern Fujian (Fig 1). Thus, it could be inferred that Chinese fir in the Taiwan area originated from north of mainland China, migrated southward during/before the glacial period via Zhejiang province and Fujian province, then, at least potentially, eastward through the overbridge across southern Fujian and Taiwan, and finally scattering as local populations. A previous report also mentioned the possibility of this route [50]. Moreover, Nantou County was considered as a major diversity center for many tree species in Taiwan [9]. In this research, genetic diversity was higher in the Miaoli population than Nantou (Table 3). Analysis of gene flow (Table 4) showed that the Miaoli population underwent frequent genetic communication with other populations, thus supporting the high level of genetic diversity. Population size (Table 1) probably contributed to that result because fewer individuals, only 4, were found in the original forest in Nantou County, thus limiting the gene pool, which provided too few polymorphic alleles statistically. Southern part of Taiwan was considered as a major refugium [51], however, unfortunately, samples from that regions haven’t been collected in this study limited the analysis of the migrant route of Chinese Fir in Taiwan.

Wild individuals were utilized as material in this study, which probably led to more reliable results. However, the limited population numbers collected in mainland China and individuals in Taiwan area probably created a negative effect. In further study, more wild populations of Chinese fir from mainland China, especially from typical geographic conditions, and larger size populations, especially in Taiwan, should be collected, and more types of molecular markers should be utilized to guarantee more accurate and reliable results.

## Conclusion

Overall, 266 polymorphic loci were developed from Chinese fir by using ISSR analysis. The results suggested different pattern of genetic diversity and variation of Chinese fir between Fujian province and Taiwan area. A possible extinct event and recolonization was revealed within Taiwan populations, whereas that might not occur within Fujian populations. The evaluation of genetic variation and structure is potentially useful for future research, which will probably focus on the evolutionary process of Chinese fir as affect by geographic events.

## Supporting information

S1 Text Present/absent information of the 266 polymorphic loci among all individuals from the ISSR amplification products. (TXT)

S1 Fig The most likely number of populations identified by the Evanno’s method. K = 7 showed the highest DeltaK value for all values of K ranging from 1 to 9.
(TIF)
