# Supplementary figures and images for: Genetic diversity and variation of Chinese fir from Fujian province and Taiwan, China, based on ISSR markers
Source: PLoS One. 2017 Apr 13;12(4):e0175571. doi: 10.1371/journal.pone.0175571 (PMC5391013; doi:10.1371/journal.pone.0175571)

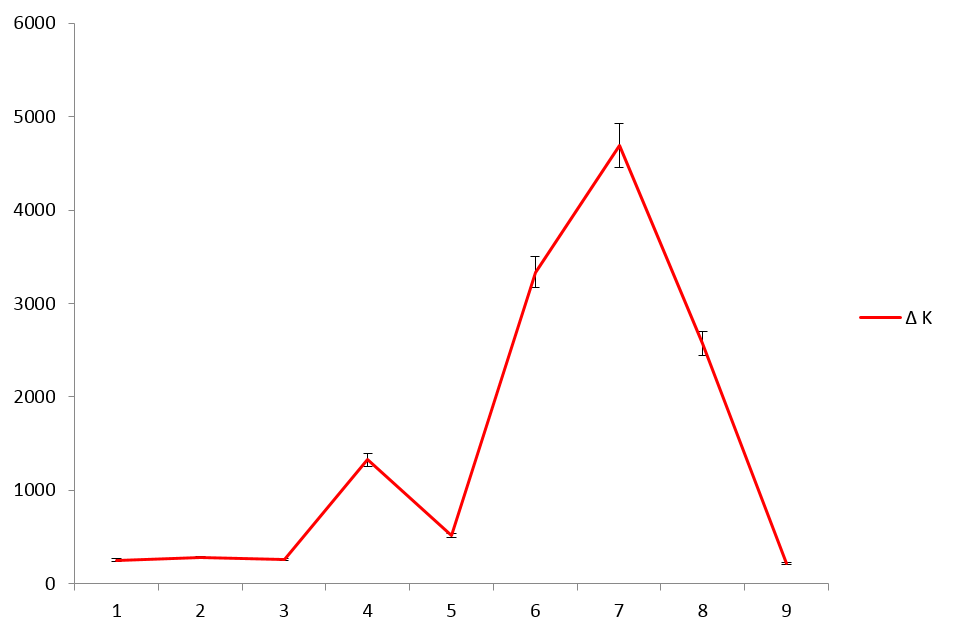

Supplement: S1 Fig — K = 7 showed the highest DeltaK value for all values of K ranging from 1 to 9. (TIF) [file pone.0175571.s002.tif]
